# Structural Guided Scaffold Phage Display Libraries as a Source of Bio-Therapeutics

**DOI:** 10.1371/journal.pone.0070452

**Published:** 2013-08-09

**Authors:** Y. K. Stella Man, Danielle DiCara, Nicole Chan, Sandrine Vessillier, Stephen J. Mather, Michelle L. Rowe, Mark J. Howard, John F. Marshall, Ahuva Nissim

**Affiliations:** 1 Centre for Tumour Biology, Barts Cancer Institute, Queen Mary's University of London, London, United Kingdom; 2 Bone and Joint Research, William Harvey Research Institute, Queen Mary's University of London, London, United Kingdom; 3 Centre for Molecular Oncology and Imaging, Queen Mary's University of London, London, United Kingdom; 4 Biological NMR Spectroscopy, School of Biosciences, University of Kent, Canterbury, Kent, United Kingdom; Vanderbilt University, United States of America

## Abstract

We have developed a structurally-guided scaffold phage display strategy for identification of ligand mimetic bio-therapeutics. As a proof of concept we used the ligand of integrin αvβ6, a tumour cell surface receptor and a major new target for imaging and therapy of many types of solid cancer. NMR structure analysis showed that RGD-helix structures are optimal for αvβ6 ligand-interaction, so we designed novel algorithms to generate human single chain fragment variable (scFv) libraries with synthetic V_H_-CDR3 encoding RGD-helix hairpins with helices of differing pitch, length and amino acid composition. Study of the lead scFv clones D25scFv and D34scFv and their corresponding V_H_-CDR3 derived peptides, D25p and D34p, demonstrated: specific binding to recombinant and cellular αvβ6; inhibition of αvβ6-dependent cell and ligand adhesion, αvβ6-dependent cell internalisation; and selective retention by αvβ6-expressing, but not αvβ6-negative, human xenografts. NMR analysis established that both the D25p and D34p retained RGD-helix structures confirming the success of the algorithm. In conclusion, scFv libraries can be engineered based on ligand structural motifs to increase the likelihood of developing powerful bio-therapeutics.

## Introduction

The use of combinatorial phage display scFv libraries for generation of therapeutic antibodies is well established and has resulted in clinically valuable reagents [1], [2]. ScFv libraries are commonly made from immune or naïve B cells or as synthetic libraries where antibody variable heavy (V_H_) and variable light (V_L_) gene segments are rearranged *in vitro* with synthetic complementarity determining regions (CDRs) coding for random sequences of varying lengths [3]–[5]. The use of the phage display library has been used to develop antibodies for therapeutic intervention using the above combinatorial libraries. We reasoned that the use of antibody engineering in combination with ligand structural studies will result in robust libraries that can lead to isolation of potent ligand-mimetic bio-therapeutic antibody candidates.

Since receptor∶ligand interactions must be considered as interacting topographical maps we wondered if it were possible to generate a target-selective library by incorporating a panel of specific three-dimensional shapes into the CDR3 of the variable heavy (V_H_-CDR3). If such a library used stereochemical shapes that corresponded to a ligand-binding interface then it would more likely generate scFv(s) that will block the ligand∶receptor interaction than would a conventional random library. To test this hypothesis we considered a therapeutically relevant target, the integrin αvβ6, which represents a novel and important tumour-selective target that is expressed on the surface of cancer cells. We, and others, have shown that αvβ6 promotes cancer cell migration, invasion and growth *in vivo* [6]–[10]. Moreover strong expression of αvβ6 correlates with poor prognosis in multiple cancers [11]–[13] and thus human therapeutic antibodies to this integrin are likely to have a significant therapeutic value.

In previous studies we identified the 20mer peptide A20FMDV2 (N_1_A_2_V_3_P_4_N_5_L_6_R_7_G_8_D_9_L_10_Q_11_V_12_L_13_A_14_Q_15_K_16_V_17_A_18_R_19_T_20_), derived from the foot and mouth disease virus VP1 coat protein, as a highly specific and potent ligand for αvβ6 [14]. The peptide included the αvβ6-binding motif RGDLXXL identified by Kraft et al (1999) [15]. Using NMR we determined the three-dimensional structure of A20FMDV2 in 30% TFE: a hairpin structure with RGD at the tip of the turn followed by a C-terminal helix [14]. This structural motif has also been observed in additional αvβ6-binding peptides (Wagstaff et al, 2012, [14]). In addition, we have previously probed the role of the helix in A20FMDV2 through the use of specific D-amino acids to disrupt helix formation, and found the resulting helix-attenuated peptide had greatly reduced binding to cells expressing αvβ6 [14].

In this study we have used our NMR data to design algorithms to build phage display human scFv libraries that would retain the key structural residues that would encode a library of RGD-helix hairpin structural motifs where the helices could vary in length, pitch and sequence composition. Our data show that it is possible to design scFv libraries that include structural motifs within the V_H_-CDR3 to provide potent ligand antagonising antibody candidates that can be developed for cancer therapy.

## Results

### Library design

**Figure 1:**
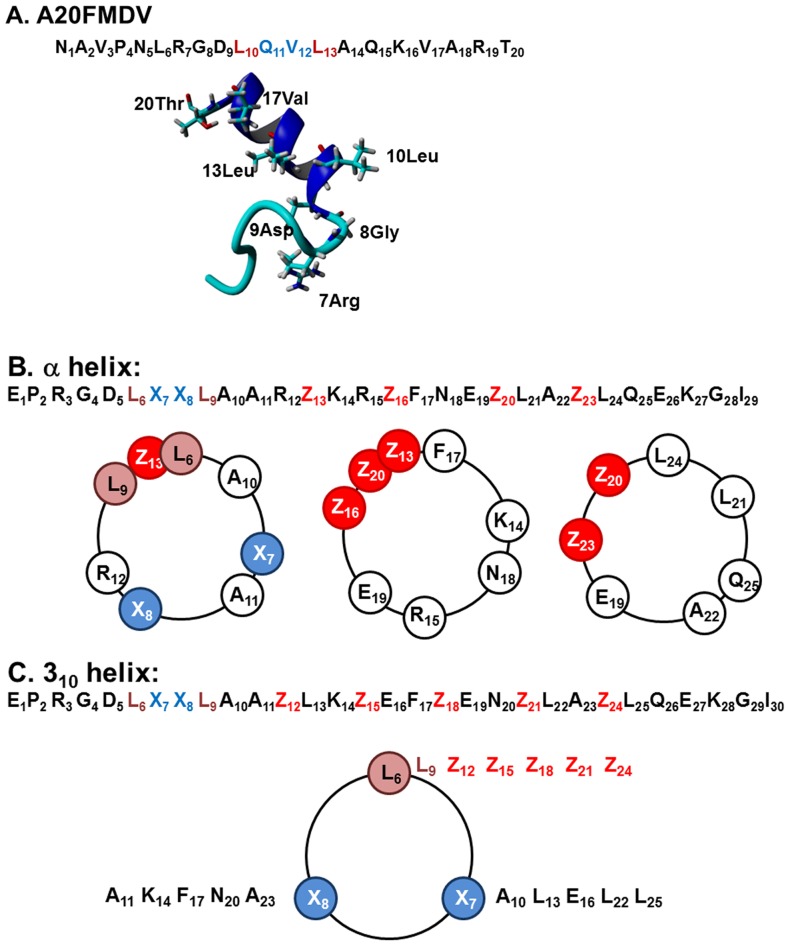
α-helix and 3_10_-helix Library design. A) Amino acid sequence and NMR solution structure of A20FMDV2 with a hairpin structure with RGD at the tip of the turn followed by a C-terminal helix is shown. B) α-helix algorithm used to design the V_H_-CDR3 encoding a hairpin containing at its turn an RGD motif, followed by a C-terminal α-helix. C) 3_10_-helix algorithm used to designed the V_H_-CDR3 encoding a hairpin containing at its turn an RGD motif, followed by a C-terminal 3_10_-helix. Amino acid positions that are available for randomisation are highlighted (X and Z shown in blue and red, respectively).

Our rationale was to introduce a structural selectivity to a phage display library where the three-dimensional (3D) αvβ6 ligand recognition motif (RGD-helix) was genetically encoded into the antibody binding pocket at the V_H_-CDR3. Figure 1A shows the amino-acid sequence identity and mean NMR solution structure of A20FMDV2. From these data plus the STD-NMR data [14] we designed two algorithms to develop V_H_-CDR3 libraries encoding a hairpin containing at its turn, an RGD motif, followed by a C-terminal α-helix (Figure 1B) or a 3_10_-helix (Figure 1C); the helical wheel map for each library is also shown. The template synthetic V_H_-CDR3 was based on the α-helix donor sequence L_8_A_9_R_10_L_11_K_12_R_13_E_14_F_15_N_16_E_17_, which is helix 1 from the Drosophila engrailed homeodomain ((EN-HD) [16]). However in the library the L_8_ was changed to A_8_ in order to prevent formation of an LXXLL motif. The α-helix template V_H_-CDR3 algorithm was: **E_1_P_2_**R_3_G_4_D_5_L_6_X_7_X_8_L_9_A_10_A_11_R_12_ **Z_13_**K_14_R_15_ **Z_16_**F_17_N_18_E_19_ **Z_20_**L_21_A_22_ **Z_23_L_24_Q_25_E_26_K_27_G_28_I_29_** where **Z** and X were random amino-acid residues introduced into the same quadrant as the leucine residues of the RGDLXXL motif: at position 13, 16, 20 and 23 in the α-helix based on the helical wheel (3.6 residues per turn of helix). The EP and the LQEKGI motifs were N- and C-terminal helix-capping regions, respectively (based on the standard Schellmann C-cap: Leu-Gln-Glu-Lys-Gly-Ile (LQEKGI) [17] (Figure 1B). To extend the helix length further, the EN-HD donor sequence was repeated from positions 21/22 in the library. To build the 3_10_ helix library we used the E_1_P_2_R_3_G_4_D_5_L_6_X_7_X_8_L_9_A_10_A_11_ **Z_12_**L_13_K_14_ **Z_15_**E_16_F_17_ **Z_18_**E_19_N_20_ **Z_21_**L_22_A_23_ **Z_24_**L_25_Q_26_E_27_K_28_G_29_I_30_ template inserting a random amino acid residue in every third position following the RGDLXXL motif in the 3_10_ helix (3.0 residues per turn of 3_10_ helix, Figure 1C). Hence, the random residues automatically provided helices of different lengths because they included helix stabilising residues, such as alanine, lysine or arginine or, alternatively, helix destroying residues such as proline [18] as well as including residues covering all side chain properties to test the effect of charge, hydrophobicity and steric interactions.

### Library selection and screening for lead candidates

**Figure 2:**
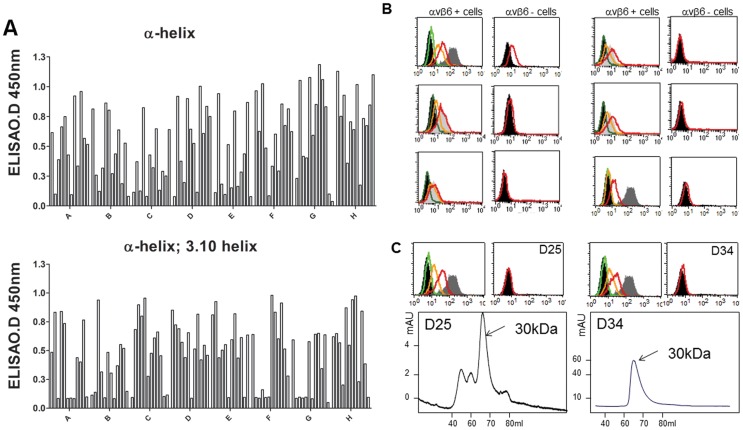
Screening of phage clones isolated from the α-helix & 3_10_-helix libraries. A) Monoclonal scFv screening ELISA testing 96 clones in each library. Bacterial supernatants were added to 5 µg/ml recombinant αvβ6 immobilized onto ELISA plate and then probed with mouse anti-Myc antibody followed by anti-mouse-HRP. B) Clones with unique sequences were screened for binding cellular αvβ6 by flow cytometry. Figure shows examples where scFv was tested at 100 (red histogram), 10 (orange histogram) and 1 (green histogram) µg/ml. For clarity the relevant αvβ6-specific mouse monoclonal antibody 10D5 (grey) and the negative control IgG (black) histograms are also shown in each plot. C) Binding to cellular αvβ6 by D25 and D34 verified by flow cytometry at 100 (red histogram), 10 (orange histogram) and 1 (green histogram) µg/ml. Size-exclusion chromatography profile of purified of D25scFv and D34scFv showed a major peaks at 30 kDa.

After 3 rounds of alternate panning on immobilised recombinant αvβ6 and cells expressing αvβ6, greater than two thirds of clones bound to recombinant αvβ6 in ELISA (Figure 2A). Binders revealed at least 24 unique V_H_-CDR3 sequences corresponding to both an α-helix and 3_10_ helix libraries. In flow cytometry experiments none of the scFv bound to the αvβ6-negative cells and many scFv bound well to the αvβ6-expressing cells. Since we used pair of cell lines that are isogenic and differ only by αvβ6 but endogenously express four other RGD directed integrins α5β1, αvβ3, αvβ5 and αvβ8 [19], [20] these data suggest strongly that the scFvs exhibited αvβ6-specific binding (Fig. 2B). However, based upon a combination of scFv protein expression yields, biochemical stability (size-exclusion chromatography) and strength of binding to cellular αvβ6 (flow cytometry) we focussed our study on 2 different scFvs clones: D25scFv (D25) and D34scFv (D34) (Figure 2C). V_H_-CDR3 sequences of both D25 and D34 encoded α-helix as follows: **EP** ***RGD***LRTLAAR***E***KR***N***FNE***T***LA***R*** **LQEKGI** and **QP** ***RGD***LRELAAR***S***EAQ**LQEKGI**, for D25 and D34, respectively. Clone D34 had mutation from C to G that resulted in replacement of E to Q in the N-terminal helix-capping regions. The size-exclusion chromatography profile of the purified D34scFv showed a main peak eluting at 70 ml, corresponding to the 30 kDa scFv. For D25scFv we also observed a lower peaks eluting at 45–60 ml, corresponding to scFv dimers and tetramers, occasionally seen with some scFv [5] (Figure 2C).

### αvβ6-specific antagonistic efficacy

**Figure 3:**
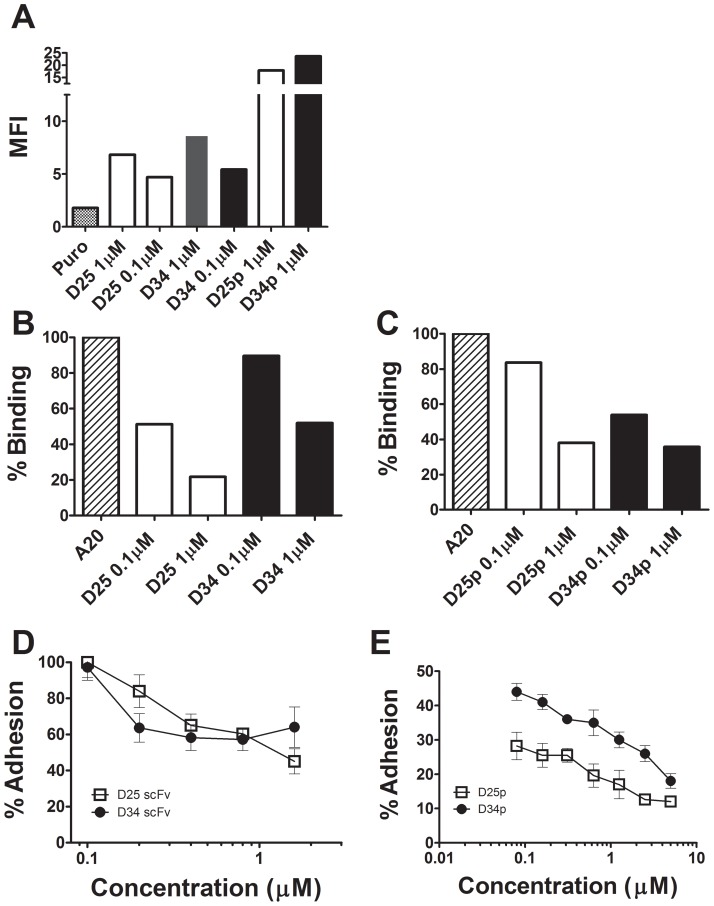
Cellular αvβ6 binding efficacy of D25scFv, D34scFv, D25p and D34p. A) Binding to A375P β6 was demonstrated by flow cytometry. The mean florescence intensity (MFI) values recorded from representative experiments are shown. A375Puro cells were used for the negative control (puro) where none of the D25scFv or D34 scFvs (data not shown) or peptide derivatives (Figure S1) showed any binding. To avoid repetition in the figure, the bar indicated as puro represent the binding of D25 scFv, D34 scFv which gave similar MFI when the primary scFv or peptide were omitted as the binding to puro control cells (data not shown). B) Dose-dependent inhibition of the αvβ6 specific binding of A20FMDV2 (A20) was demonstrated with both D25 (D25scFv) and D34 (D34scFv). A20FMDV2 binding is expressed here as a percentage of the MFI value detected in the absence of scFv. C) Dose-dependent inhibition of the αvβ6 specific binding of A20FMDV2 (A20) was demonstrated with D25p and D34p peptides. A20FMDV2 binding is expressed here as a percentage of the MFI value detected in the absence of peptide inhibitors. D) αvβ6-dependent adhesion to fibronectin was similarly inhibited by D25scFv and D34scFv (p>0.05). E) αvβ6-dependent adhesion to fibronectin was inhibited by D25p and D34p. Significantly better inhibition of αvβ6-dependent adhesion to fibronectin was seen by D25p in comparison to D34p (p = 0.0156).

A dose dependent binding analysis of both D25scFv and D34scFv and their corresponding V_H_-CDR3 peptides (D25p and D34p, respectively) exhibited αvβ6-specific binding to A375Pβ6 cells but not A375Ppuro (Figure 3A and Figure S1). Additionally, D25scFv, D34scFv, D25p and D34p showed a dose-dependent inhibition of binding to cellular αvβ6 of A20FMDV2 peptide (NAVPNLRGDLQVLAQKVART), a peptide that is 1000-fold more selective for αvβ6 over α5β1, αvβ3, αvβ5 and αvβ8 integrins and binds with high affinity (K_D_ 1.7 nM) to the αvβ6 integrin [19] (Figure 3B–C). Moreover, D25scFv, D25p, D34scFv and D34p exhibited significant concentration-dependent inhibition of αvβ6-dependent adhesion of carcinoma cells to immobilised fibronectin (Figure 3D–E). Inhibition by D25p was significantly stronger than D34p (p = 0.0156)

### αvβ6-specific internalization

**Figure 4:**
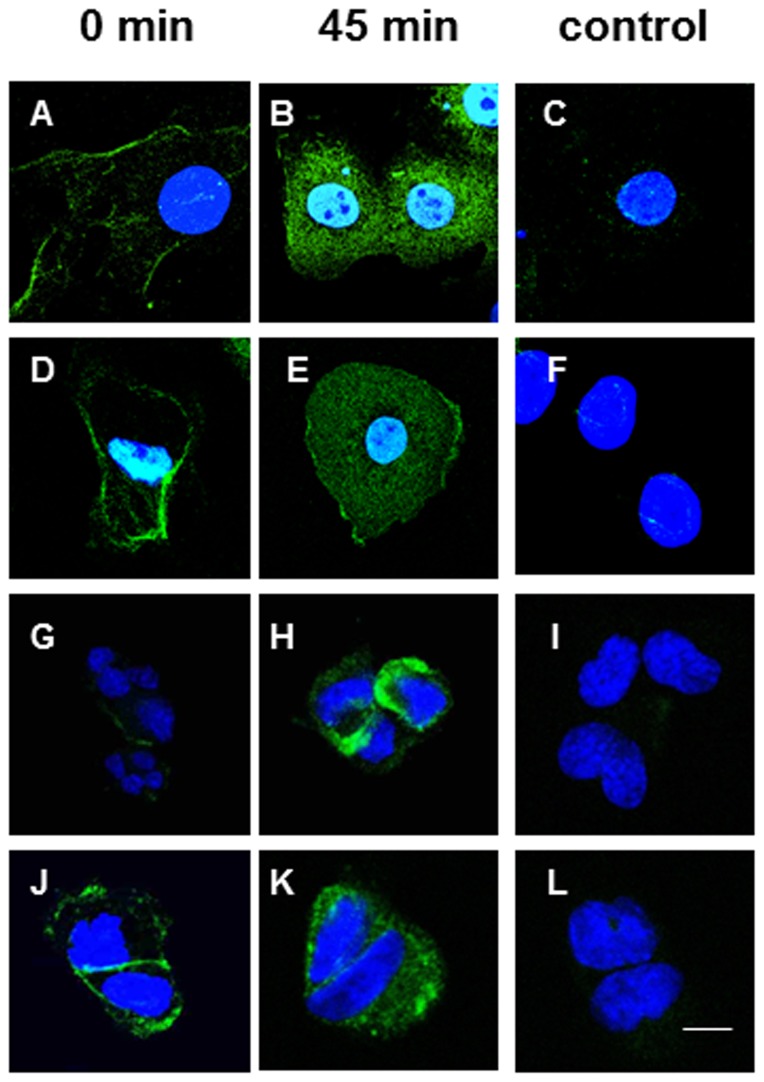
Internalisation of D25scFv, D34scFv, D25p and D34p in αvβ6-expressing cells. Internalisation of bound D25scFv (A,B) and D34scFv (D, E) was assessed at 0 mins (A,D) and 45 mins (B,C,E,F) in αvβ6-expressing cells (A,B,D,E) and detected using anti-myc antibody. In control cells (C,F) only anti-myc antibody was used. The scFvs both were internalised by αvβ6-expressing cells and some located to the nucleus. The absence of nuclear staining with anti-myc antibody alone suggests this was a true nuclear localisation. Internalisation of D25p (G, H, I) and D34p (J, K, L) was assessed in αvβ6-expressing cells ((G,H,J,K) and αvβ6-negative cells (I, L) at the times indicated. Both peptides were internalised only by αvβ6-expressing cells. The scale bar shown represents 20 µm.

D25scFv and D34scFv exhibited cellular internalisation in αvβ6-expressing cells but not in αvβ6-negative cells. At 0 minutes, the scFvs were localised at the cell surface (Figure 4A and 4D for D25scFv and D34scFv, respectively) but after incubation at 37°C for 45 minutes, they were localised within the cell cytoplasm and nucleus (Figure 4B and 4E for D25scFv and D34scFv, respectively). Omitting the scFv primary layer and labelling only with anti-myc and the fluorochrome-labelled secondary antibody showed very little nuclear staining suggesting the nuclear localisation of the scFvs was real (Figure 4C and 4F for D25scFv and D34scFv, respectively). Similarly, biotinylated-D25p and biotinylated-D34p also underwent cellular internalisation in αvβ6-expressing cells (Figure 4H and 4K for D25p and D34p, respectively) but not in αvβ6-negative cells (Figure 4I, and 4L for D25p and D34p, respectively). Efficient internalisation was observed at 30–45 minutes (Figure 4H, K for D25p and D34p, respectively) but no nuclear localisation was observed.

### Biotinylated-D25 peptide localises to αvβ6-expressing tumours *in vivo*

**Figure 5:**
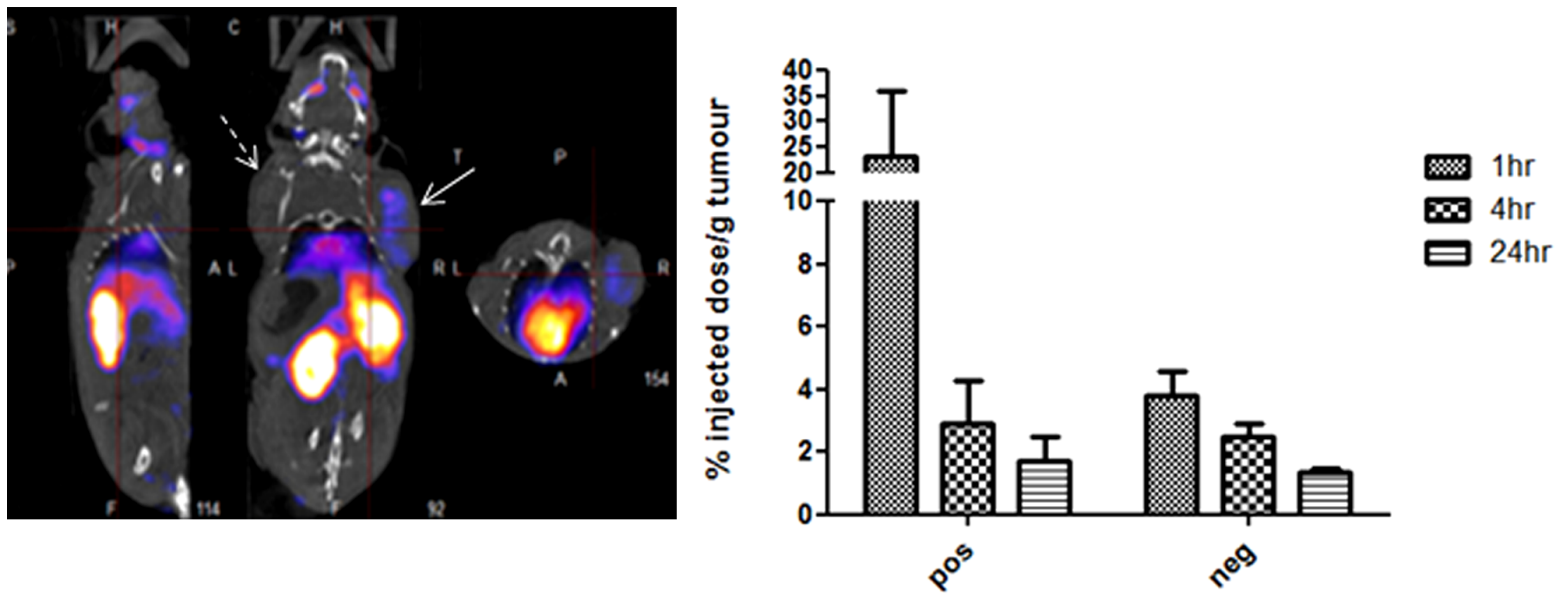
Localization of radiolabelled D25p *in vivo*. Single Photon Emission Computed Tomography imaging was used to localize the Indium-111 radiolabelled D25p *in vivo*. The figure in the left panel represents a representative mouse at the one-hour time point, post injection. The three images represent three different viewing angles – the sagittal (left), coronal (middle) and axial (right). Significantly more radioactivity was retained by the αvβ6 positive tumour (indicated with solid arrow) compared with the αvβ6 negative tumour (indicated with the dashed arrow). Quantitative data showing the average retention of radioactivity per gram of tumour in the three tested mice at the 1 h, 4 h and 24 h time points are shown in the histogram. Significantly more radioactivity was retained by the αvβ6 positive tumour (p<0.01) compared with the αvβ6 negative tumour, and significantly higher at 1 hr compare to 4 and 24 hr (p<0.01). No significant difference were seen in radioactivity retention in the αvβ6 negative tumour between 1 hr and 4 and 24 hr in the (p>0.05).

When 12.5MBq of [In111]-DTPA-Streptavidin decorated with biotinylated-D25p was intravenously injected into three mice bearing both a subcutaneous αvβ6-positive A375Pβ6 tumour and an αvβ6-negative A375Ppuro tumour, on opposite shoulders, we observed a 23% injected dose per gram uptake in the αvβ6-positive tumour compared with only 3% for the αvβ6-negative tumour at 1 hour post-injection, a ratio of almost 8∶1 (Figure 5). This translated into a very clear discrimination of the αvβ6-positive tumour by single-photon emission computed tomography (SPECT) imaging (Figure 5).

### Structural determination of D25 and D34 peptides by NMR

**Figure 6:**
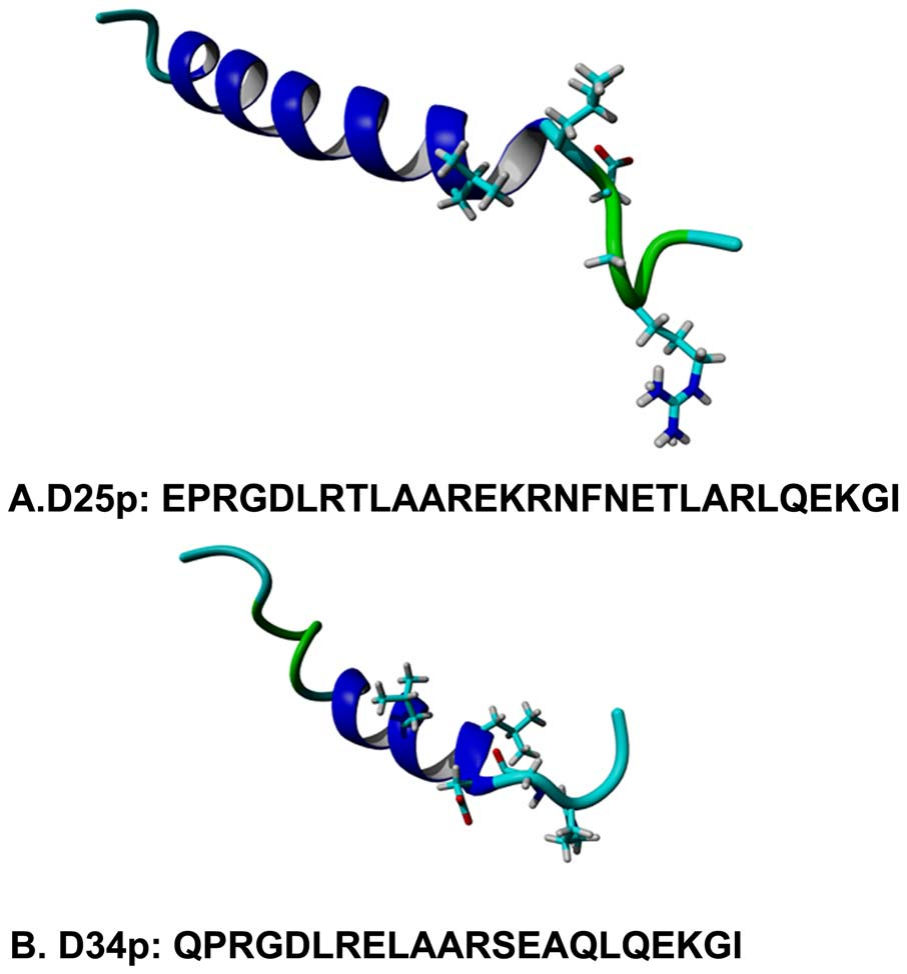
NMR solution Structure of D25p and D34p. Figure shows the 3D-rendering model for each peptide based on the mean of 20 NMR structures. Both peptides exhibited the RGD-helix motif. D34 which has 22 amino-acids has a shorter helix than peptide D25, which has 29 amino-acids. Helices for both peptides were defined as standard α-helix with the D34 α-helix running from Leu6-Leu17 and D25 α-helix running from Leu6-Gln25.

We used NMR to solve the solution structures for peptides D34p and D25p. Figure 6 shows the 3D-rendering model closest to the mean calculated for each peptide from an ensemble of 20 NMR structures; the associated structural statistical data from CNS for both peptides is available in Table S1 and NOE and structural contact information is available in Figures S2, S3, S4. Both peptides exhibited the RGD-helix motif. D34 which has 22 amino-acids has a shorter helix than peptide D25, which has 29 amino-acids. Helices for both peptides were defined as standard α-helix with the D34 α-helix running from Leu6-Leu17 and D25 α-helix running from Leu6-Gln25.

## Discussion

CDR grafting, the replacing of complementarity-determining regions in a mouse antibody with those from a human antibody, has been used for more than two decades for humanization of mouse monoclonal antibodies and development of immunotherapy [20]. CDR grafting of ligands into CDR loops to bind to a specific receptor was originally reported by Barbas *et al* in 1993 where they produced semisynthetic human antibodies library that included RGD motifs followed by random sequences to select for antibody fragments specific to the integrins αvβ5, αvβ3, and α_I_IIbβ3 [21]. Kogelberg *et al* inserted 17 residues from A20FMDV2 into the CDR3 region into an anti-CEA scFv thereby creating an antibody with αvβ6-specificity [22]. More recently, a peptide sequence that bound to an inorganic material surface, was grafted into the CDR of a camel-type single domain antibody rearranged with a library of random sequences in additional CDR. Authors noted a synergistic effect from the grafted and selected random CDR loops that drastically increased the affinity for the inorganic target [23]. In this study we have taken a different approach, namely, grafting a 3-dimensional geometry based library designed from a ligand∶receptor binding stereochemical interface. To test the model we chose a therapeutically valuable target, the integrin αvβ6 that we, and others, have reported is associated with poor survival from cancer, presumed to be because this integrin promotes carcinoma invasion and survival [6]–[9]. We had identified previously αvβ6-binding peptides from high affinity ligands for αvβ6 and shown that interrogation of the peptide structures by various NMR techniques revealed 1) all three ligands (A20FMDV1, LAP, A20FMDV2) were hairpin-shaped peptides with RGD at the turn followed by an helix and 2) the Asp+1 and Asp+4 residues were exposed on the same face of the helix and appeared to form a hydrophobic binding interface with the integrin and 3) potency of αvβ6 inhibition appeared to correlate with the length of the helix [14], [24]. Thus we designed two algorithms to retain these structural elements while allowing for variation in amino-acid composition and helix length.

We have used our NMR data to design algorithms that would retain the key structural residues that would encode a library of RGD-helix-hairpin structural motifs where the helices would be of varying lengths and sequence composition. We used the algorithms to create two structurally-guided scFv libraries that incorporate either an α- or a 3_10_-helix C-terminal to the RGDLXXL motif within V_H_-CDR3.

Screening of 96 clones isolated following three rounds of biopanning with the combined α-helix and 3_10_-helix libraries revealed H-CDR3 sequences of both α-type and 3_10_-type, indicating that both library designs are capable of producing αvβ6-binding scFv. The scFv and the V_H_-CDR3 derived peptides from the two lead clones, D25scFv, D34scFv, D25p and D34p: 1) bound only to αvβ6-expressing cells (A375Pβ6) but not to cells that expressed αvβ3, αvβ5, αvβ8 and α5β1 (A375Ppuro) 2) exhibited dose-dependent inhibition of the αvβ6-specific ligand A20FMDV2 binding to cellular αvβ6 3) inhibited carcinoma cell αvβ6-dependent adhesion to fibronectin 4) and were internalised into cells in an αvβ6-dependent manner. These characteristics make these two lead clones excellent candidates for development as therapeutic antibodies. In advance of this we tested whether the D25p possessed similar *in vivo* targeting capabilities that we had previously shown for A20FMDV2. Data showed that the radiolabelled D25p selectively located to αvβ6-expressing tumours *in vivo* showing that the structurally designed library could generate unique compounds with *in vivo* efficacy and specificity. Our attempts to radiolabel the D25scFv and D34scFv resulted in loss of antibody activity so *in vivo* experiments were not attempted. This will need to be addressed in future studies where radiolabeling protocols will need to be modified. Hence antibody derivatives such as diabody and intact antibody derivatives of D25 and D34 could be studied further in an *in vivo* setting.

Our data also show that RGD-helix structures with very long helices retain excellent αvβ6-specificity and function-blocking activity. Thus D25p has an α-helix almost 3-fold longer than the parental peptide A20FMDV2. Interestingly, the D25p was a more efficient inhibitor of αvβ6-cellular function than D34p which has a shorter α-helix, whereas there was no difference in binding of D34scFv versus D25scFv to cellular αvβ6 (Figure 3E). Thus, while we have not established whether there is an optimal length of α-helix for scFv specificity for αvβ6, we have established that relatively large 3D-motif-encoding sequences can be grafted into CDR loops and result in function-blocking antibodies and V_H_-CDR3 based peptides.

The concept introduced here can be adopted for other therapeutic targets. Current studies have identified tumour associated receptor tyrosine kinases that are being considered as potential therapeutic targets [25]–[27] and it is likely that key chemokine receptors [28] also will be similarly targeted. A similar strategy to that described here, using the 3-dimensional shape of the ligand-binding interface for these receptors, can generate receptor-selective structurally-guided scFv, peptide or any other protein scaffold libraries that would herald a new method for creating valuable therapeutic antibodies. This approach has the potential to replace the traditional approach whereby random libraries are used to developed ligand antagonists and may result in more powerful therapeutic antibodies.

## Materials and Methods

### Library construction and selection

**Figure 7:**
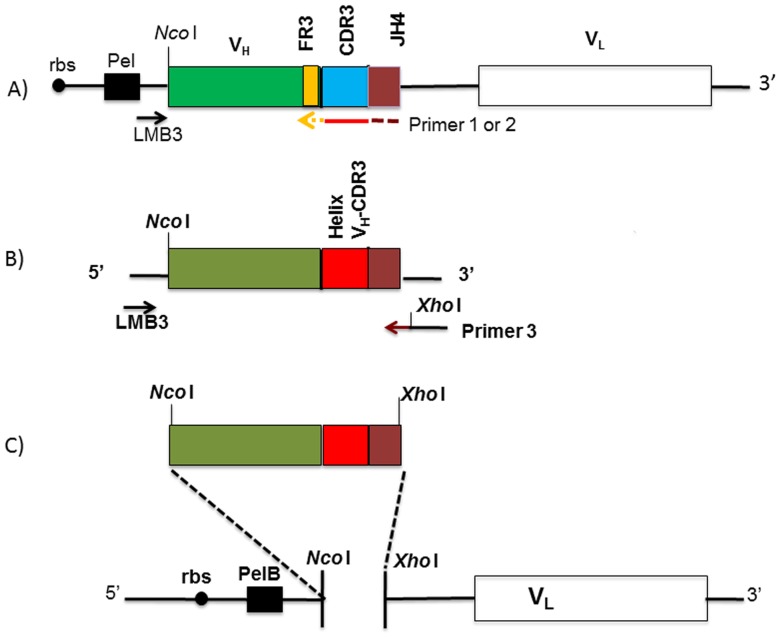
Cloning strategy used to build the scFv phage display libraries with synthetic structurally shaped V_H_-CDR3 containing the α- and 3_10_-helix. A. pHEN1 plasmid backbone containing scFv was used as the PCR template gene to be amplified with the primers LMB3 and the Primer 1 or 2 that includes the FR3 sequences (yellow), flanked by the α- or 3_10_-helix algorithms (red) and followed by JH4 sequences (brown). B. After PCR amplification the resulted V_H_ gene segment was rearranged with α- or 3_10_-helix V_H_-CDR3 (red). To incorporate a unique Xho1 restriction site, the PCR products derived from step A were amplified with LMB3 and Primer 3. C. The resultant PCR products from step B were digested with Nco1 and Xho1 for insertion into the pIT2 plasmid backbone that contain light chain repertoire.

Library construction is shown in Figure 7. A pool of 50 human V_H_ genes cloned into the pHEN1 vector was used as a PCR template [5]. The library initially was amplified with LMB3 primer (Table 1), which anneals to pHEN1 vector sequences 5′ to the cloned V_H_ gene and primer 1or 2, which anneals to 3′ end of the V_H_ gene which was composed of the frame work 3 (FR3) region, the structurally guided motifs encoded by the α- or 3_10_ -helix algorithms and finally the JH4 sequences (Table 1, Fig. 7A). In a second PCR step, XhoI restriction site was introduced 3′ to the JH4 sequence after amplification with primer 1 or 2 and LMB3 (Figure 7B). The V_H_ gene amplicons containing inserts encoding the algorithms were then digested with XhoI and NcoI restriction enzymes and inserted into the NcoI and XhoI treated pIT2 vector containing the VL repertoire (Fig. 7C, [3]).

**Table 1:** Oligonucleotides used to build the structural guided library.

Primer I.D	Sequence (5′ to 3′)
**LMB3**	CAGGAAACAGCTATGAC
**Primer 1/α-helix**	CCAGATCCCTTTCTCCTGCAAMNNGGCTAGMNNCTCGTTGAAMNNCCGCTTMNNTCGGGCTGCGAGMNNMNNTAGGTCTCCTCGAGGTTCTCTTGCACAGTAATACACGGCCGTGTC
**Primer 2/3_10_-helix**	CCAGATCCCTTTCTCCTGCAAMNNCGCGAGMNNGTTCTCMNNGAACTCMNNCTTCAGMNNGGCTGCGAGMNNMNNTAGGTCTCCTCGAGGTTCTCTTGCACAGTAATACACGGCCGTGTC
**Primer 3**	GCCTGAACCGCCTCCACCACTCGAGACGGTGACCAGGGTACCTTGGCC-CCAGATCCCTTTCTCCTGCAA
**Fdseq**	GAATTTTCTGTATGAGG

The α-helix and 3_10_ libraries were each rescued separately KM13 helper phage as described previously [29]. For selection, we used in parallel each library separately as well as mixed alpha helix and 3_10_ libraries. The initial phage selection was performed using immobilised recombinant αvβ6 protein as described previously [5], [29] and http://www.lifesciences.sourcebioscience.com/ media/143421/tomlinsonij.pdf. After two rounds of selection on immobilised αvβ6 we then selected the libraries on cell-expressed αvβ6 using A375Pβ6 and A375PPuro cell lines, for αvβ6-positive and αvβ6-negative selection, respectively [14], [22]. First line screening were done by ELISA using 5 µg/ml immobilised recombinant αvβ6 as described [29].

### Cell lines

The adherent melanoma cell lines A375Pβ6 and A375Ppuro, described previously [22] were cultured in Dulbecco's Modified Eagles' Medium (DMEM) supplemented with 10% Foetal Calf Serum (FCS). The human oral squamous cell carcinoma VB6, which was engineered to express high levels of αvβ6, was grown in Keratinocyte Growth Medium as described [7].

### Screening for integrin specificity by Flow Cytometry

Purified scFv were tested for specificity for αvβ6 by flow cytometry using the isogenic pairs of cell lines, A375Pβ6 and A375Ppuro, or DX3β6 and DX3puro. Each pair expresses similar levels of four RGD-binding integrins (αvβ3, αvβ5, αvβ8, α5β1) but only A375Pβ6 and DX3β6 express αvβ6 [14], [22], [30]. Briefly, cells were detached with trypsin/EDTA, rinsed twice in ice-cold wash buffer (0.1% BSA/DMEM/0.1% NaN_3_) and approximately 2×10^5^ cells re-suspended in 50 µl of wash buffer per sample. In between all incubation steps, all samples were washed twice with wash buffer unless otherwise stated. Cells were incubated on ice for 1 hr with 0.1 or 1 uM purified scFv of peptide, washed twice. For scFv, cells were then incubated for 1 hr on ice with mouse anti-Myc antibody (clone 9E10, Santa Cruz) at 1∶100. After washing, bound antibody was detected with anti-mouse IgG-Alexaflour 488 (1∶250 for 1 h; Molecular Probes), on ice. Bound V_H_-CDR3 derived peptide were detected by rabbit anti-biotin IgG (1∶200), followed by anti-rabbit IgG-Alexafluor 488 (1∶250; Molecular Probes). Cells were analysed using the FACSCalibur (Beckton Dickinson). Cell expression of αvβ6 integrin was detected with mouse monoclonal antibody (clone 10D5, 10 µg/ml; Millipore) and non-specific (control) binding with non-immune class matched IgG.

### Inhibition of αvβ6 ligand binding

The scFv lead candidates propensity to inhibit binding of the αvβ6-specific biotinylated-A20FMDV2 [19] to αvβ6-expressing cells was determined by pre-incubation with purified scFv or peptide (0.1 and 1.0 µM) for 10 mins, immediately followed by 40 mins incubation with the biotinylated A20FMDV2 (10 nM). Bound A20FMDV2 peptides were detected by rabbit anti-biotin IgG (1∶200), followed by anti-rabbit IgG-Alexafluor 488 (1∶250; Molecular Probes).

### Fast protein liquid chromatography (FPLC)

The structural stability of scFv proteins was assessed by gel filtration chromatography using a HiPrep 16/60 sephacryl S-200 (GE healthcare, Amersham, UK) connected to an AKTA FPLC (GE healthcare, Amersham, UK). Fractionations were performed in PBS at a flow rate of 0.3 ml/min with detection at 280 nm. A Calibration Kit (GE healthcare, Amersham, UK) containing protein markers (aprotinin, ribonuclease A, carbonic anhydrase, ovalbumin and conalbumin) was used to generate a calibration curve. Equal amounts of each of the protein markers were prepared to 500 µl in PBS prior to loading onto the column. Kav values derived from the equation: Kav = (V_e_-V_0_)/(V_c_-V_0_); where Ve = elution volume, Vc = geometric column volume, and V0 = column void volume were plotted against log molecular weight to generate a protein calibration curve. Purified scFv (50 ug/500 ul) was loaded onto the gel filtration column and molecular weight of the different peaks was determined using the calibration curve.

### Internalisation Assay

In 24-well culture plates, 2×10^4^ cells αvβ6-positive and –negative cells were seeded onto 13 mm diameter glass coverslips and allowed to incubate overnight at 37°C in growth medium. Cells were washed twice in serum free medium (SFM) and then scFv (4 uM) or biotinylated-CDR3-peptide (100 nM) diluted in SFM added on ice for 10 mins. After two ice-cold washes and a further 10′ on ice with mouse anti-biotin antibody (10 ug/ml) pre-warmed media containing 10% FCS was added to the cells and incubated at 37°C. At 10 min intervals between 0–60 mins, cells were fixed in 2% HCHO in PBS and permeabilised with 0.1% TritonX-100 (PBS) for 3 mins. scFv was detected with mouse anti c-myc (1∶100 dilution, clone 9E10; 30′, ambient temperature) while peptides were detected by rabbit anti-biotin IgG (1∶200). Both peptide and scFv detected with anti-mouse Alexa488 for 30′. Nuclei were counter-labelled with 4′,6-diamidino-2-phenylindole (DAPI), coverslips mounted with Mowiol and samples examined by confocal microscopy (Zeiss LSM510, Welwyn Garden City).

### Picogreen adhesion assay

All adhesion assays were performed in quadruplicate and repeated at least 2–3 times. In a 96-well plate format, test wells were coated with Fibronectin (10 ug/ml/PBS) and negative control wells were coated with 0.1% BSA (bovine serum albumin)/PBS. After incubation at 37°C for 1 hour, plates were washed in PBS twice and blocked with 0.1% BSA/PBS for 30 mins at 37°C. Following a PBS rinse, cells were seeded into the wells, the plate resting on ice to avoid dehydration and to ensure an initial uniform temperature for the experiment. Purified scFv or peptide was added (25 ul) to the wells at the desired concentrations, before adding 1.5×10^4^ cells (25 ul) per well. In some experiments, cells (1.5×10^4^) were pre-treated with β1 blocking antibody AIIB2 at 10 ug/ml before seeding into test wells to block the β1-dependent fibronectin adhesion. To determine percentage adhesion, standard curves were generated by plating 0–2.5×10^4^ cells in separate wells. After incubating plates at 37°C for 30′ plates were washed twice in 1 mM CaCl_2_/0.5 mM MgCl_2_/PBS and transferred into −80°C for 15 mins. Adherent cells were quantified using a Picogreen kit (Invitrogen) and analysed on a fluorescence reader (FLUOstar Optima, BMG Labtech Ltd, Bucks, UK).

### *In vivo* localization

All animal procedures followed strict Home Office (UK) guidelines under license number PPL 80/2279. Biotinylated-peptide was radiolabelled with Indium-111. To 10 ug of DOTA-Biotin (Macrocyclics.com #C-100) buffered in 1M Ammonium Acetate (pH 5.5) was added Indium [^111^In]-acetate. The mixture was heated to 80°C for 30 mins and cooled at RT for 10 mins. The labelled DOTA-Biotin was added to streptavidin at a 1∶1 Molar Ratio (1 mg of streptavidin per 17.6 ug of DOTA-Biotin). To this mixture, 88 ug of botinylated-D25p was added and 10 ul of the mixture analysed by size exclusion-HPLC to verify the stability of radiolabelled products pre- and post-labelling (data not shown). A total of 50MBq was used to label 25 ug of peptide; the sample was divided into four such that each mouse received 12.5 MBq (6.25 ug) of ^111^In-labelled D25 peptide.

Female CD1 nu/nu athymic nude mice (Charles River) were subcutaneously injected with 100 µl (2×10^6^ cells) of A373Pβ6 into the right shoulder and A375puro in the left shoulder. Tumours were allowed to develop for 20 days and 200 µl of freshly radiolabelled ^111^In-labelled D25 peptide was administered intravenously. Tumours were imaged by NanoSPECT/CT (Bioscan, Inc) at 1 hr, 4 hr and 24 hr-time points as follows: Mice were placed onto the imaging bed and initial low resolution CT scans were collected at 45KVp, 180 projections per rotation, 500 ms per projection. Subsequently SPECT images were acquired (45 minutes acquisition time) and data reconstructed using on-board HiSPECT-NG software (Bioscan). To measure radioactivity associated with tumours the NanoSPECT/CT machine was calibrated (before the experiment) by imaging a phantom with an Indium-111 standard solution. Subsequently Invivoscope software (Invicro) was used to generate three-dimensional regions of Interest (ROI) around the tumours and the ROIs converted to megabequerels.

### Structural determination by NMR study

Solution NMR structures of peptides D25p and D34p on 0.1%TFE were solved as previously (13). All NMR data for peptides A22 (D25p) and A29 (D34p) were obtained at 283 K from a 14.1 T (600 MHz ^1^H) Bruker Avance III NMR spectrometer equipped with a 5 mm QCI-F cryoprobe. All NMR samples were 350 µL within a Shigemi NMR tube and contained 1 mM peptide in 25 mM sodium phosphate buffer at pH 6.5 also containing 50 mM sodium chloride, 4% (v/v) dimethylsulfoxide (DMSO), and 30% (v/v) trifluroethanol-d3 (TFE). NMR data processing was completed using TopSpin 3.1 (Bruker), assignments were completed using CCPN Analysis.

^1^H chemical shifts and through-space structural assignments were obtained from two-dimensional TOCSY and NOESY NMR experiments with mixing times of 20 ms/60 ms for TOCSY and 250 ms for NOESY. The observed NOE contacts support the presence of an α-helical conformation along the length of the peptide with NOEs observed between Hα and HN (i–i+3) as well as Hα and Hβ (i–i+3). Structural ensembles were calculated using CNS [31] and including dihedral angles confirmed by DANGLE analysis [31] and predicted hydrogen-bond donor acceptor pairs. The final ensemble was water-minimised using YASARA Structure software (available from http://www.yasara.org) and Ramachandran analysis of each peptide ensemble was completed using PROCHECK-NMR [32].

### Statistical analysis

The Wilcoxon matched-pairs signed rank test was used to compare activity. Statistical analysis was performed using the GraphPad Prism software package (GraphPad Software, San Diego, CA).

## Supporting Information

Figure S1 A375Ppuro cells were incubated with biotinylated D25p or D34p at 1 uM. Bound peptide was detected with rabbit anti-biotin IgG (1∶200), followed by anti-rabbit IgG-Alexafluor 488 (1∶250; Molecular Probes). Note that there was no significant binding to the cells by either peptide (clear histograms). Black histograms represent controls where peptides were omitted.
(TIF)

Figure S2 **Structures of RGD peptides D34p (A+B) and D25p (C+D) in 30% w/v TFE.** RGD residues are shown as sticks in A and C. Helices are drawn for and ensembles are fitted to residues 6–17 and 6–25 for peptide D34p and D25p respectively.
(TIF)

Figure S3 **NOE contacts, chemical shift difference, hydrogen bond donors and Dihedral restraints for D34p peptide with 30% w/v TFE.** The secondary structure shown beneath the restraints indicates the limits of helix formation according to Ramachandran analysis of the final 20 structure ensemble.
(TIF)

Figure S4 **NOE contacts, chemical shift difference, hydrogen bond donors and Dihedral restraints for D29p peptide in 30% w/v TFE.** The secondary structure shown beneath the restraints indicates the limits of helix formation according to Ramachandran analysis of the final 20 structure ensemble.
(TIF)

Table S1 **NMR and refinement statistics for 20 structure ensembles of peptides.**
(DOCX)
