# Supplementary figures and images for: Structural Guided Scaffold Phage Display Libraries as a Source of Bio-Therapeutics
Source: PLoS One. 2013 Aug 9;8(8):e70452. doi: 10.1371/journal.pone.0070452 (PMC3739823; doi:10.1371/journal.pone.0070452)

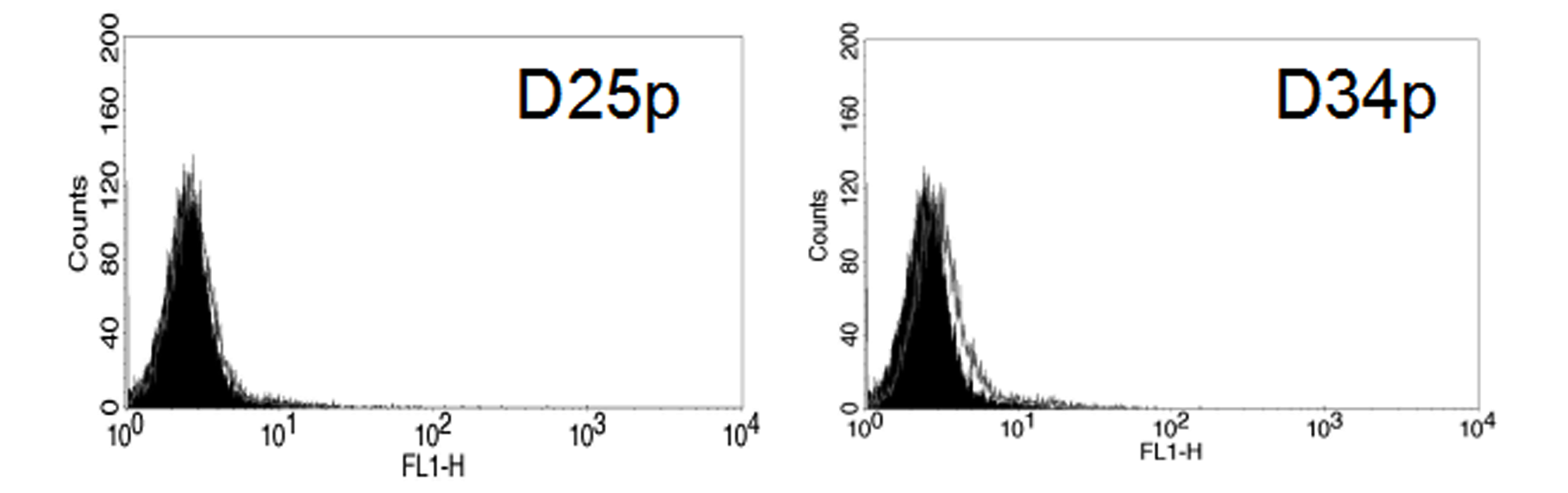

Supplement: Figure S1 — A375Ppuro cells were incubated with biotinylated D25p or D34p at 1 uM. Bound peptide was detected with rabbit anti-biotin IgG (1∶200), followed by anti-rabbit IgG-Alexafluor 488 (1∶250; Molecular Probes). Note that there was no significant binding to the cells by either peptide (clear histograms). Black histograms represent controls where peptides were omitted. (TIF) [file pone.0070452.s001.tif]

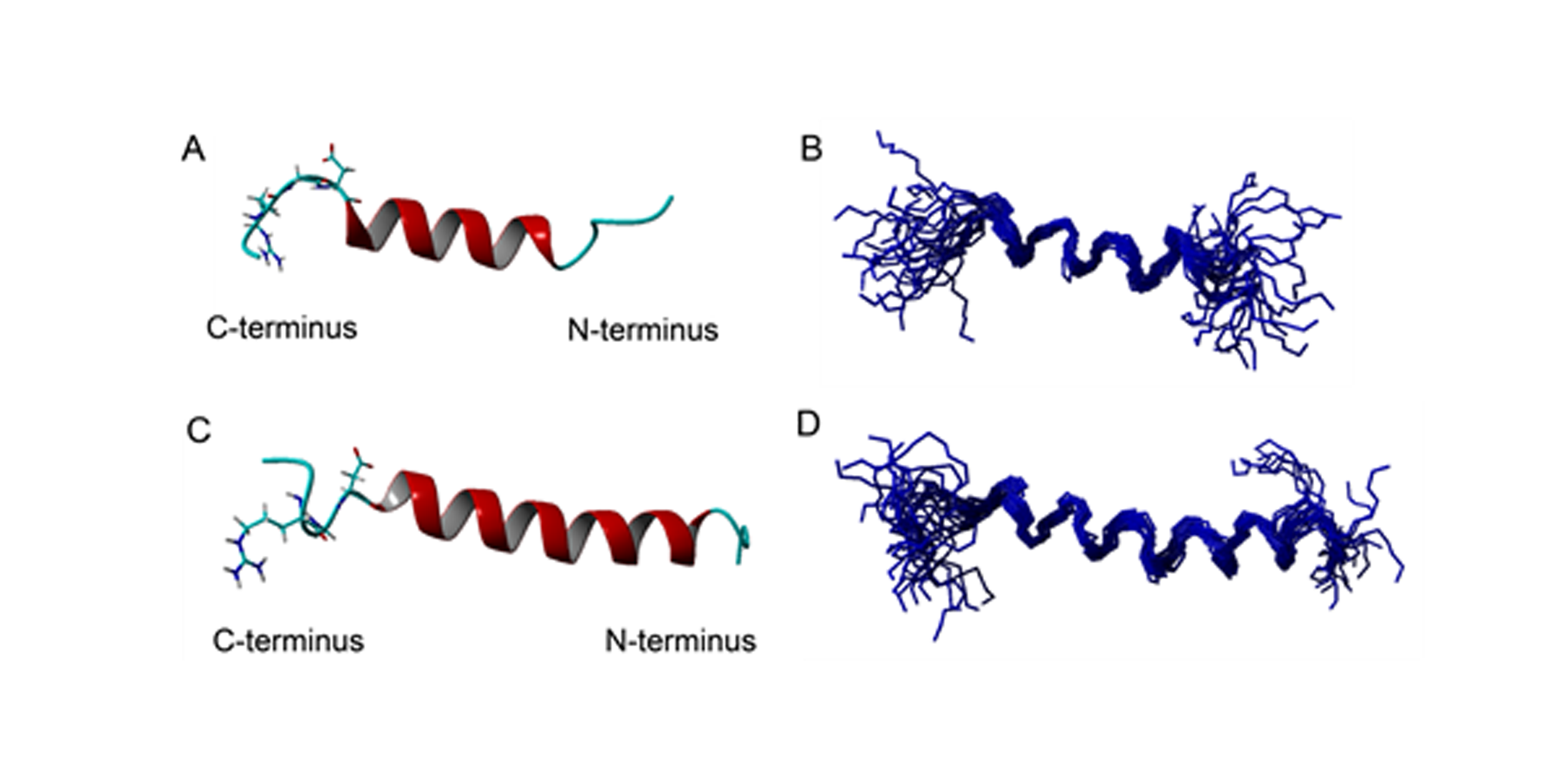

Supplement: Figure S2 — Structures of RGD peptides D34p (A+B) and D25p (C+D) in 30% w/v TFE. RGD residues are shown as sticks in A and C. Helices are drawn for and ensembles are fitted to residues 6–17 and 6–25 for peptide D34p and D25p respectively. (TIF) [file pone.0070452.s002.tif]

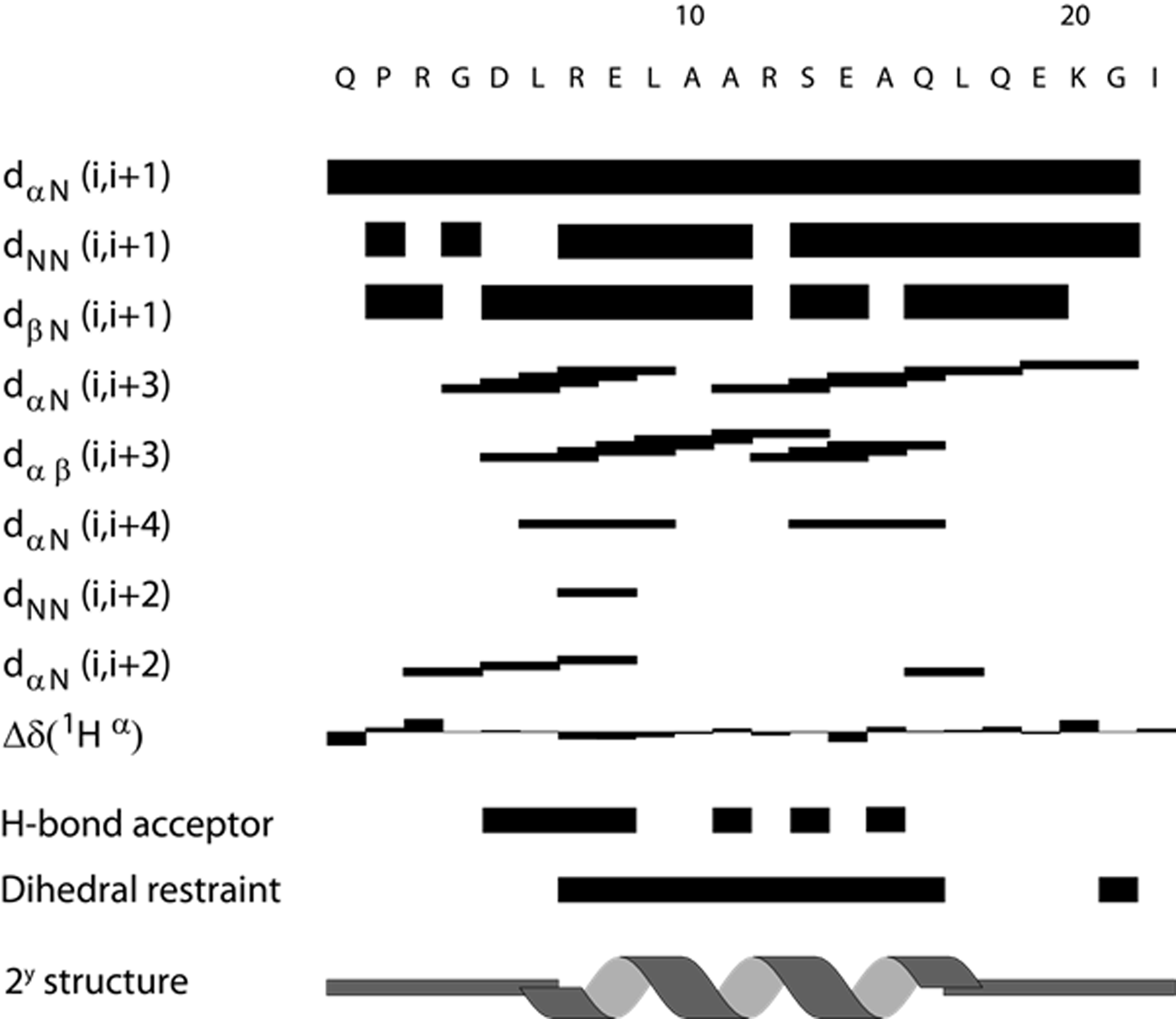

Supplement: Figure S3 — NOE contacts, chemical shift difference, hydrogen bond donors and Dihedral restraints for D34p peptide with 30% w/v TFE. The secondary structure shown beneath the restraints indicates the limits of helix formation according to Ramachandran analysis of the final 20 structure ensemble. (TIF) [file pone.0070452.s003.tif]

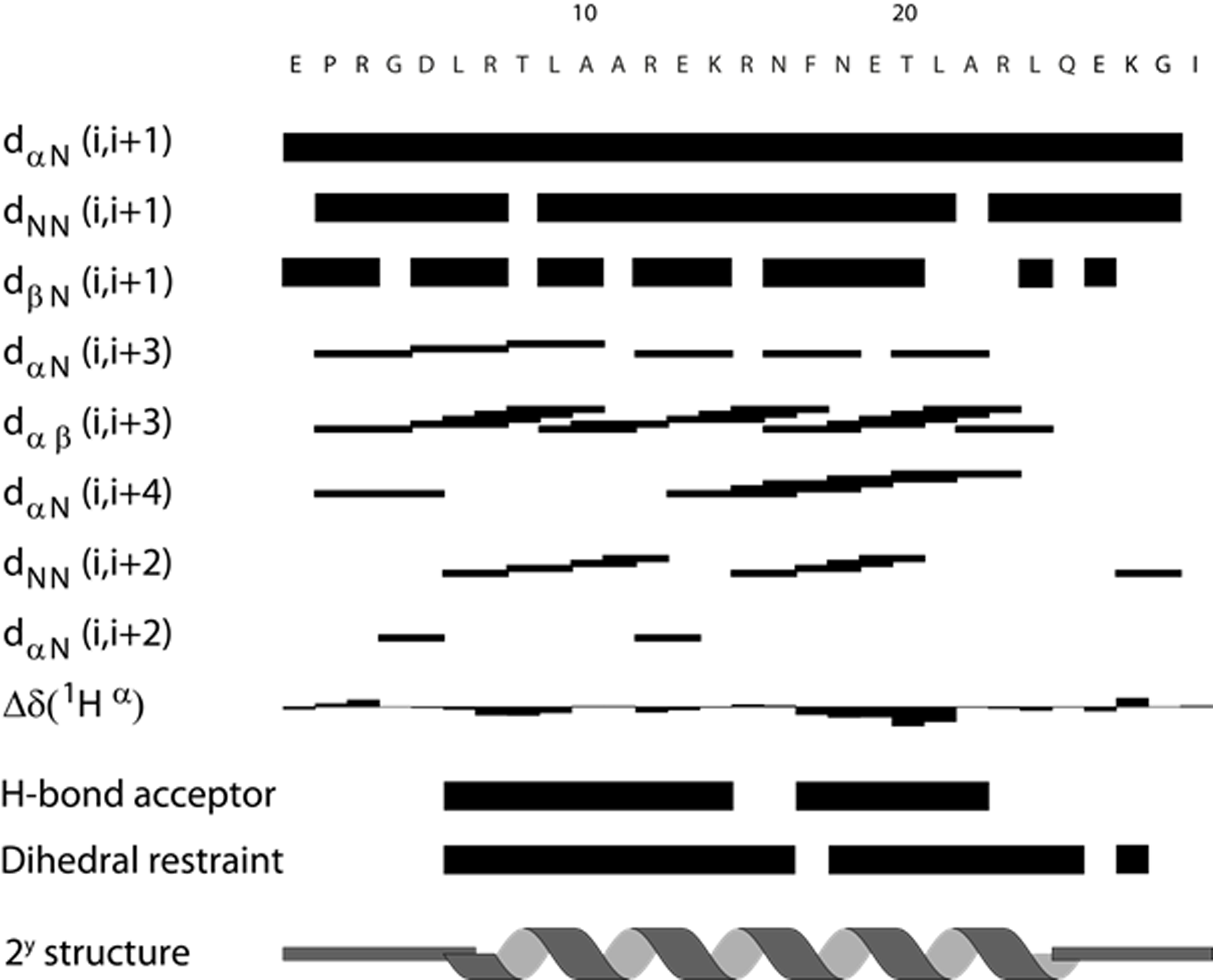

Supplement: Figure S4 — NOE contacts, chemical shift difference, hydrogen bond donors and Dihedral restraints for D29p peptide in 30% w/v TFE. The secondary structure shown beneath the restraints indicates the limits of helix formation according to Ramachandran analysis of the final 20 structure ensemble. (TIF) [file pone.0070452.s004.tif]
