# Supplementary material for: Structural Guided Scaffold Phage Display Libraries as a Source of Bio-Therapeutics
Source: PLoS One. 2013 Aug 9;8(8):e70452. doi: 10.1371/journal.pone.0070452 (PMC3739823; doi:10.1371/journal.pone.0070452)
Supplement: Table S1 — NMR and refinement statistics for 20 structure ensembles of peptides. (DOCX) [file pone.0070452.s005.docx]

**Table S1.** **NMR and refinement statistics for 20 structure ensembles of peptides**

|  | **D34p** | **D25p** |
| --- | --- | --- |
| **NMR distance and dihedral constraints** |  |  |
| Distance constraints |  |  |
| Total NOE | 319 | 624 |
| Intra-residue | 115 | 233 |
| Inter-residue | 204 | 391 |
| Sequential (\|*i* – *j*\| = 1) | 123 | 200 |
| Medium-range (\|*i* – *j*\| < 4) | 79 | 189 |
| Long-range (\|*i* – *j*\| > 5)  Hydrogen bonds  Dihedral restraints | 2  7  22 | 2  15  40 |
| Lennard-Jones Energy (kJ mol^-1^)  **Structure statistics*** | -890.97 | -1608.67 |
| Violations |  |  |
| NOE violations >0.2 Å | 0 ± 0.002 | 0 ± 0.007 |
| Dihedral angle violations >2.0 ° | 0 ± 0.008 | 0 ± 0.007 |
| Ramachandran (%)† |  |  |
| Most favoured regions | 85.0 | 88.1 |
| Additionally allowed regions | 10.9 | 10.2 |
| Generously allowed regions | 3.5 | 1.0 |
| Disallowed regions | 0.6 | 0.6 |
| Average pairwise r.m.s. deviation* (Å) | Over residues 6-17 | Over residues 6-25 |
| Heavy | 1.346 | 1.431 |
| Backbone | 0.540 | 0.649 |

*For all accepted structures of 40 structure calculation

† For 20 lowest energy water minimised structures
